# Characterization and applications of glutaminase free L-asparaginase from indigenous *Bacillus halotolerans* ASN9

**DOI:** 10.1371/journal.pone.0288620

**Published:** 2023-11-28

**Authors:** Ifrah Shafqat, Shaheen Shahzad, Azra Yasmin, Muhammad Tausif Chaudhry, Safia Ahmed, Aneela Javed, Imran Afzal, Monaza Bibi

**Affiliations:** 1 Genomics Research Lab, Department of Biological Sciences, International Islamic University Islamabad, Islamabad, Pakistan; 2 Microbiology and Biotechnology Research Lab, Department of Biotechnology, Fatima Jinnah Women University, Rawalpindi, Pakistan; 3 National Physical and Standards Laboratory, Islamabad, Pakistan; 4 Department of Microbiology, Faculty of Biological Sciences, Quaid-i-Azam University, Islamabad, Pakistan; 5 Atta-ur-Rahman School of Applied Biosciences (ASAB), National University of Sciences and Technology (NUST), Islamabad, Pakistan; 6 Department of Biology, Lahore Garrison University, Lahore, Pakistan; Konkuk University, REPUBLIC OF KOREA

## Abstract

L-asparaginase (L-ASNase) is a versatile anticancer and acrylamide reduction enzyme predominantly used in medical and food industries. However, the high specificity of L-asparaginase formulations for glutamine, low thermostability, and blood clearance are the major disadvantages. Present study describes production, characterization, and applications of glutaminase free extracellular L-asparaginase from indigenous *Bacillus halotolerans* ASN9 isolated from soil sample. L-asparaginase production was optimized in M9 medium (containing 0.2% sucrose and 1% L-asparagine) that yielded maximum L-ASNase with a specific activity of 256 U mg^-1^ at pH 6 and 37°C. L-asparaginase was purified through acetone precipitation and Sephadex G-100 column, yielding 48.9 and 24% recovery, respectively. Enzyme kinetics revealed a *V*max of 466 mM min^-1^ and *K*m of 0.097 mM. Purified L-ASNase showed no activity against glutamine. The purified glutaminase free L-ASNase has a molecular mass of 60 kDa and an optimum specific activity of 3083 U mg^-1^ at pH 7 and 37°C. The enzyme retains its activity and stability over a wide range of pH and temperature, in the presence of selected protein inhibitors (SDS, β-mercaptoethanol), CoCl_2_, KCl, and NaCl. The enzyme also exhibited antioxidant activity against DPPH radical (**IC**_50_ value 70.7 μg mL^-1^) and anticancer activity against U87 human malignant glioma (**IC**_50_ 55 μg mL^-1^) and Huh7 human hepatocellular carcinoma (**IC**_50_ 37 μg mL^-1^) cell lines. Normal human embryonic kidney cells (HEK293) had greater than 80% cell viability with purified L-ASNase indicating its least cytotoxicity against normal cells. The present work identified potent glutaminase free L-ASNase from *B*. *halotolerans* ASN9 that performs well in a wide range of environmental conditions indicating its suitability for various commercial applications.

## Introduction

L-asparaginase (L-ASNase, E.C.3.5.1.1) is a commercially important enzyme that catalyzes the hydrolysis of L-asparagine (L-ASN) into L-aspartic acid and ammonia [1]. There are two types of L-asparaginase; type I and type II [2]. The type I asparaginases have affinity for both L-glutamine and L-asparagine. Whereas, only Type II L-asparaginase have anticancer activity and have higher specificity towards L-asparagine [3]. It is commonly found in animals, plants, fungi, and bacteria [4, 5]. The enzyme is primarily used in the treatment of acute lymphoblastic leukemia (ALL), hematological and non-hematological disorders [6]. It is also used in food industry to neutralize carcinogenic acrylamide and as a biosensor to detect asparagine levels during chemotherapy [7].

L-asparaginase targets cancer cells by starving them of asparagine. Cancer cells lack asparagine synthetase and hence cannot carry out de novo synthesis of asparagine. Thus, L-ASNase activity causes depletion of asparagine in blood, resulting in the inhibition of protein synthesis for cancer cells [8]. Five commercial formulations of anti-leukemic drug (L-ASNase) are available for ALL treatment: three formulations are based on *E*. *coli* asparaginase (Elspar®; Leukanase; Kidrolase); a pegylated form of *E*. *coli* asparaginase (Oncaspar®); and recombinant asparaginase from *Erwinia chrysanthemi* (Erwinase®) [9].

L-ASNase is also a promising acrylamide mitigating agent that reduces carcinogenic acrylamide production in food industry. The carcinogenic acrylamide is produced through the Maillard reaction when starch-rich foods (potato fries, cookies) are treated above 100°C [10]. Currently, two commercial L-ASNase (Acrylaway and PreventAse) are available for processing starch-rich food at the industrial level [9]. Different challenges are associated with the current applications of this versatile enzyme. One of the major challenge is in the pharmaceutical application where glutaminase activity of asparaginase results in hypersensitivity reactions in ALL patients [11]. Similarly, L-ASNase is a thermolabile enzyme that hinders its application in food sector; PreventAse and Acrylaway can process food ingredients only at 50°C and 37°C, respectively [9]. The inevitable use of L-ASNase in pharmaceutical as well as food industry and constraints associated with the commercially available formulations require exploration of new sources of L-ASNase with an improved activity that could be produced through commercially viable processes [12]. Bacterial L-asparaginases are much easier to extract and purify, boosting their production and application in the industries [13]. Considering this demand of glutaminase free L-ASNase with improved biochemical and enzymatic properties for therapeutic applications, this research was carried out to isolate an indigenous bacterial strain from environmental samples for L-ASNase production having no glutaminase activity. The enzyme was purified and biochemically characterized for its optimal functioning. The purified enzyme was also assessed for its substrate specificity, anticancer and antioxidant activity.

## Materials and methods

### Isolation and characterization of L-asparaginase producing bacteria

For isolation of L-ASNase producing bacteria, ten soil and nine water samples were collected from different cities of Pakistan. All the samples were serially diluted and spread on nutrient agar plates. After incubation at 37°C for 24 h, morphologically different colonies were selected. For qualitative screening of L-ASNase producing bacteria, colonies were streaked on modified M9 minimal medium containing (g L^-1^) Na_2_HPO_4_.2H_2_O (6 g), KH_2_PO_4_ (3 g), NaCl (0.5 g), 1M MgSO_4_.7H_2_O (2 mL), 0.1 M CaCl_2_.2H_2_O (1 mL), glucose (0.2%), agar (2%), supplemented with 1% L-asparagine and 0.009% (w/v) phenol red as indicator [14]. For initial screening of L-ASNase producing bacteria, pH of L-asparagine supplemented agar medium was kept acidic (6). After 48 h of incubation at 37°C, colonies having pink zone around them were selected for further studies [15]. Morphological, biochemical, and molecular characterization of the selected strain was carried out through standard Gram’s staining, biochemical tests and 16S rRNA gene sequencing, respectively. Gene sequence was submitted to the GenBank database and accession number was obtained.

### Production and quantification of extracellular L-asparaginase through submerged shake flask method

Extracellular L-ASNase production was carried out using modified M9 minimal medium containing (g L^-1^) Na_2_HPO_4_.2H_2_O (6 g), KH_2_PO_4_ (3 g), NaCl (0.5 g), 1M MgSO_4_.7H_2_O (2 mL), 0.1 M CaCl_2_.2H_2_O (1 mL), glucose (0.2%), and 1% L-asparagine as substrate [14]. Nutrient broth inoculated with a loopful of fresh culture was incubated for 24 h at 37°C in orbital shaker at 75 rpm. 1% of inoculum was transferred to 100 mL of M9 medium. After every 24 h of incubation at 37°C, cells were centrifuged at 12298 *g* at 4°C for 10 min. Supernatant was taken as crude enzyme and enzyme quantification assay was carried out using Nessler’s method till sixth day of incubation [16]. In the first step, crude enzyme (500 μL) was added in 500 μL of 0.4 mM L-asparagine prepared in 50 mM Tris-HCl buffer (pH = 7.40). All the contents were vortex mixed and incubated at 37°C for 30 min. Reaction was terminated by adding 500 μL of 1.5 M trichloroacetic acid. In the second step, about 100 μL of previous reaction mixture, Nessler’s reagent (100 μL) and 3.75 mL of distilled water was added and incubated at 37°C for 10 min in water bath. The absorbance was measured at 450 nm against the control. Enzyme units were interpreted in international unit. One international unit of enzyme is defined as the amount of enzyme required to produce 1 micromoles of ammonia per min. Standard curve of ammonia from ammonium chloride was prepared for calculating units of enzyme [17].


$${\text{Units}\;\text{mL}}^{-1}=\;\frac{\text{Micromoles}\;\text{of}\;\text{ammonia}\;\text{liberated}\;\times \;\text{X}}{\text{V}\;\times \;\text{T}\;\times \;\text{Y}}$$

Where X = initial volume (mL) of enzyme mixture of the first step

Y = volume of enzyme mixture (mL) (taken from first step) used in the second step

T = incubation time (min)

V = volume of enzyme taken as crude enzyme (mL).

### Optimization of L-asparaginase from *Bacillus halotolerans* ASN9 under submerged fermentation

Submerged fermentation of L-ASNase was carried out in M9 modified medium supplemented with 1% L-asparagine. For the optimization of L-ASNase production, different factors were optimized including pH (6, 7, 8), temperature (30, 37, 44°C), incubation time (day 1–6), 0.2% carbon sources (glucose, sucrose, starch), and 1% nitrogen source (L-asparagine, L-asparagine + yeast extract, L-asparagine + peptone). Each optimum parameter determined for enzyme productivity was applied for further studies.

### Optimization of *B*. *halotolerans* ASN9 L-asparaginase production by response surface methodology

Box-Behnken design of response surface methodology (RSM) was utilized to investigate the influence of different factors (pH, temperature and incubation time) on the production of L-asparaginase and determine the most appropriate levels of these factors for the maximum production of L-asparaginase.

A matrix composed of 17 experiments was used to evaluate the optimized conditions for production of *B*. *halotolerans* ASN9 L-ASNase. The matrix included three replicas at the center point to estimate the experimental error; evaluate the quadratic, linear, and interaction effects of variables; and adjust a second-order model with quadratic terms. The L-ASNase (U mg^-1^) produced was chosen as the response value, and the factors of pH (A), temperature (B) and incubation time (C) were selected as independent variables. The actual and coded values of the independent variables are shown in Table 1. Analysis of variance (ANOVA, p<0.05) was used to determine the statistical significance of the developed model. The standardized effects were used to analyze regression coefficients (based on t test, p<0.05). The coefficient of determination (R^2^), adjusted coefficient of determination (R^2^-adj) were used to assess the model’s quality. The data was analyzed and graphics were created using Design-Expert 13 software.

**Table 1:** Variables and levels of Box–Behnken planning for optimization of L-ASNase production by *B*. *halotolerans* ASN9.

Symbol	Factor	Unit	Minimum	Maximum	Coded low	Coded high
**A**	pH		6	8	-1 ↔ 6	+1 ↔ 8
**B**	Temperature	˚C	30	44	-1 ↔ 30	+1 ↔ 44
**C**	Incubation time	Days	1	6	-1 ↔ 1	+1 ↔ 6

### Purification of L-asparaginase

Crude extract of L-asparaginase produced from *Bacillus halotolerans* ASN9 was precipitated using different concentrations (40–80%) of chilled acetone. Precipitated protein was suspended in 50 mM Tris-HCl buffer at pH 7.4 [18]. Sample prepared in previous step was loaded on pre-equilibrated Sephadex G-100 column with 50 mM Tris-HCl buffer at pH 7.4 [19]. Protein elution was done with the same buffer. Total 25 fractions were collected (3 mL each). The fractions were then assayed for enzyme activity. Chromatographic run was monitored for protein at 280 nm. Molecular mass of purified L-ASNase was estimated by 7.5% SDS-PAGE according to Laemmli [20]. Proteins were stained with Coomassie Blue and then destained in distilled water. Protein size was calculated using GelAnalyzer version 19.1.

### Biochemical characterization of L-asparaginase

#### Effect of pH and temperature on activity and stability of L-asparaginase

The effect of pH and temperature on enzyme activity was studied using a range of pH (4–10) and temperature (10–80°C). pH was set using different buffers of 50 mM concentration: acetate buffer (pH 4–5), phosphate buffer (pH 6), Tris-HCl buffer (7–9), and Glycine-NaOH buffer (10). Assay conditions were maintained as mentioned above with buffers of specific pH and temperatures. Stability of the enzyme at different pH was determined by pre-incubating the enzyme with 50 mM buffers of pH 4–10 for 30 min at 37°C. Stability of L-ASNase at different temperatures was determined by pre-incubating the enzyme at 10–80°C, pH 7 for 30 min. Effect of incubation time on the stability of purified L-ASNase was determined by mixing 0.25 mL of purified enzyme with 0.25 mL of Tris-HCl buffer of pH 7 (optimal pH) and incubating at 37°C (optimal temperature) for different intervals. Sample (0.04 mL) was drawn at different time intervals (0, 0.5, 1, 2, 4, 8, 16, 24, 30, 36, 48 h) and L-ASNase activity was estimated through standard assay. The enzyme assay was performed with 0.4 mM L-asparagine. Results were expressed in terms of residual activity, which is the percentage of treated enzyme activity to that of untreated one.

#### Effect of increasing concentration of NaCl and metal salts on activity and stability of L-asparaginase

Effect of different concentrations of NaCl on L-ASNase activity was determined by incubating enzyme with substrate in the presence of different concentrations of NaCl (2 to 20%). Stability was determined by pre-incubating the purified L-ASNase with different concentrations of NaCl at 37°C for 30 min. Rest of the assay conditions were kept same. Effect of different metal ions on enzyme activity was evaluated in the presence of 5 mM of different monovalent and divalent metal ions including Na_2_CO_3_, MgSO_4_, CuSO_4_, CoCl_2_, NiCl_2_, KCl, NaCl, FeS, CaCl_2_.2H_2_O, ZnCl_2_ and MnSO_4_. Stability of enzyme in presence of metal salts was determined by pre-incubating the purified L-ASNase with 5 mM metal salts at 37°C for 30 min. Enzyme activity was measured at 37°C, pH 7. The activity was considered 100% in the absence of these salts. Residual activity was determined as mentioned before.

#### Effect of various inhibitors surfactants and solvents on activity of L-asparaginase

One mM concentration of EDTA, β-mercaptoethanol, triton-X100, tween-20, tween 80, SDS and organic solvent (0.1% DMSO, 50% ethanol and 50% isopropanol) were used to study their effect on enzyme activity. Results were expressed in terms of residual activity.

#### Substrate specificity and kinetics of L-asparaginase

The affinity of L-ASNase towards urea, L-glutamine, and L-asparagine was compared by replacing 0.4 mM L-asparagine with urea or glutamine as the substrate. The activity was determined as discussed previously in enzyme assay. The kinetics of purified enzyme were determined by non-linear regression from Michaelis Menten plot with L-asparagine (0.1–1.4 mM) as substrate using the software GraphPad prism5 [21].

### Biotechnological applications of L-asparaginase

#### Antioxidant activity of glutaminase free L-asparaginase (Free radical scavenging activity using DPPH procedure)

For the assessment of antioxidant activity of purified L-ASNase, free radical scavenging activity was reported using DPPH [21]. Different concentration (20–120 μg mL^-1^) of purified L-apsaraginase were prepared in buffer. Then, mixture of 5 μL of test sample and 95 μL of DPPH were incubated at 37°C under dark conditions for one hour. Optical density was then recorded at 517 nm in microtiter plate. The concentration of the enzyme required to scavenge 50% of DPPH (**IC**_50_) was estimated through linear regression of equation obtained after plotting of percentage scavenging activity against concentrations. Optical density of blank with only DPPH was taken as control. Percentage Scavenging was measured by applying the following formula:

$$\text{Percentage}\;\text{Scavenging}\;=\;\frac{\;Absorbance\;of\;Control\;-\;Absorbance\;of\;Sample\;}{\;Absorbance\;of\;Control}\;\times \;100$$

**IC**_50_, half maximum inhibitory concentration of the enzyme was also calculated.

#### MTT (3-(4, 5-dimethylthiazol-2-yl)-2, 5-diphenyltetrazolium bromide) assay for anticancer activity

Anticancer activity of purified L-ASNase was evaluated in vitro by using U87 cells derived from a human malignant glioma and Huh7 cell lines derived from a hepatocellular carcinoma through MTT assay. In order to determine the effect of purified L-ASNase on viability of normal cells; HEK293 (immortalized human embryonic kidney cells) cell line as used. Cells were cultured for cell line activity in Dulbecco modified eagle media (DMEM)–high glucose with 10% FBS (Thermo Fischer Scientific, Waltham, MA, USA) and 1% pen-strep MTT (3-[4, 5-dimethylthiazol-2-yl]-2,5-diphenyltetrazolium bromide (Sigma-Aldrich, St. Louis, MO, USA). Exponentially growing cells (10,000 cells per well) were plated, in triplicate, in flat-bottomed 96-well plates (Nunc, Roskilde, Denmark). Purified L-ASNase (5, 10, 15, 20, and 30 μg mL^-1^) was added to the 96-well plate to obtain a final volume of ∼200 μL per well and incubated at 37°C for 48 h. Negative control and standard anti-cancer drug Doxorubicin (DoX) were also treated in triplicates. After incubation, 5 mg mL^-1^ of MTT was dissolved in 1 mL PBS and 15 μL of prepared MTT solution was added to each well and incubated for 3 h at 37°C. Following the formation of formazan crystals, all the solution from each well was removed. Solubilizing solution (DMSO) was added to each well. The plates were left at room temperature for few min. The absorbance of the cells was recorded at 550 nm. Experiments were performed in triplicates [22].

## Results

The current studies focus was on the isolation of L-ASNase producing bacteria for optimized enzyme production, purification, characterization, and biotechnological applications.

### Screening and isolation of L-asparaginase producing bacterial isolates

L-ASNase producing bacteria were isolated from different soil and water samples and screened for L-ASNase production. Twenty L-ASNase producing bacterial isolates were compared for their ability to produce extracellular L-ASNase using a qualitative assay. Five isolates (ASN4, ASN9, ASN12, ASN14, ASN38) that produced largest hydrolytic zones were selected (S1 Table and S1A Fig). These five strains were then quantitatively assessed for L-ASNase production (S1B Fig). Among them, ASN9 produced maximum enzyme activity of 153 U mg^-1^ on third day when grown in M9 medium supplemented with 1% L-asparagine at pH 6 and 37°C under shaking condition. Therefore, only ASN9 was selected for further characterization.

### Identification of L-asparaginase producing bacterial isolate

Gram’s staining identified ASN9 cells as Gram-positive bacilli arranged in chains. Biochemical characterization revealed that ASN9 was a catalase positive strain that could also hydrolyze ONPG (o-nitrophenyl-β-D-galactopyranoside), Esculin, Arabinose, Xylose, Cellobiose, Sucrose, Trehalose and Glucose. ASN9 showed negative results for lysine, ornithine, urea, citrate, phenylalanine, malonate, adonitol, rhamnose, melibiose, raffinose, tryptophan and pyruvate/creatine hydrolysis. Molecular identification based on 16S rRNA gene sequence revealed highest similarity (98.94%) of ASN9 to *Bacillus halotolerans*, when compared against GenBank database (S2 Fig). Hence, the isolate was named as *Bacillus halotolerans* ASN9 (Accession number MZ882157.1).

### Production of L-asparaginase from *Bacillus halotolerans* ASN9

Optimization of parameters for L-ASNase production from *Bacillus halotolerans* ASN9 was performed in M9 medium supplemented with 1% L-asparagine.

### Effect of pH, temperature, carbon and nitrogen source on L-asparaginase production

**Fig 1:**
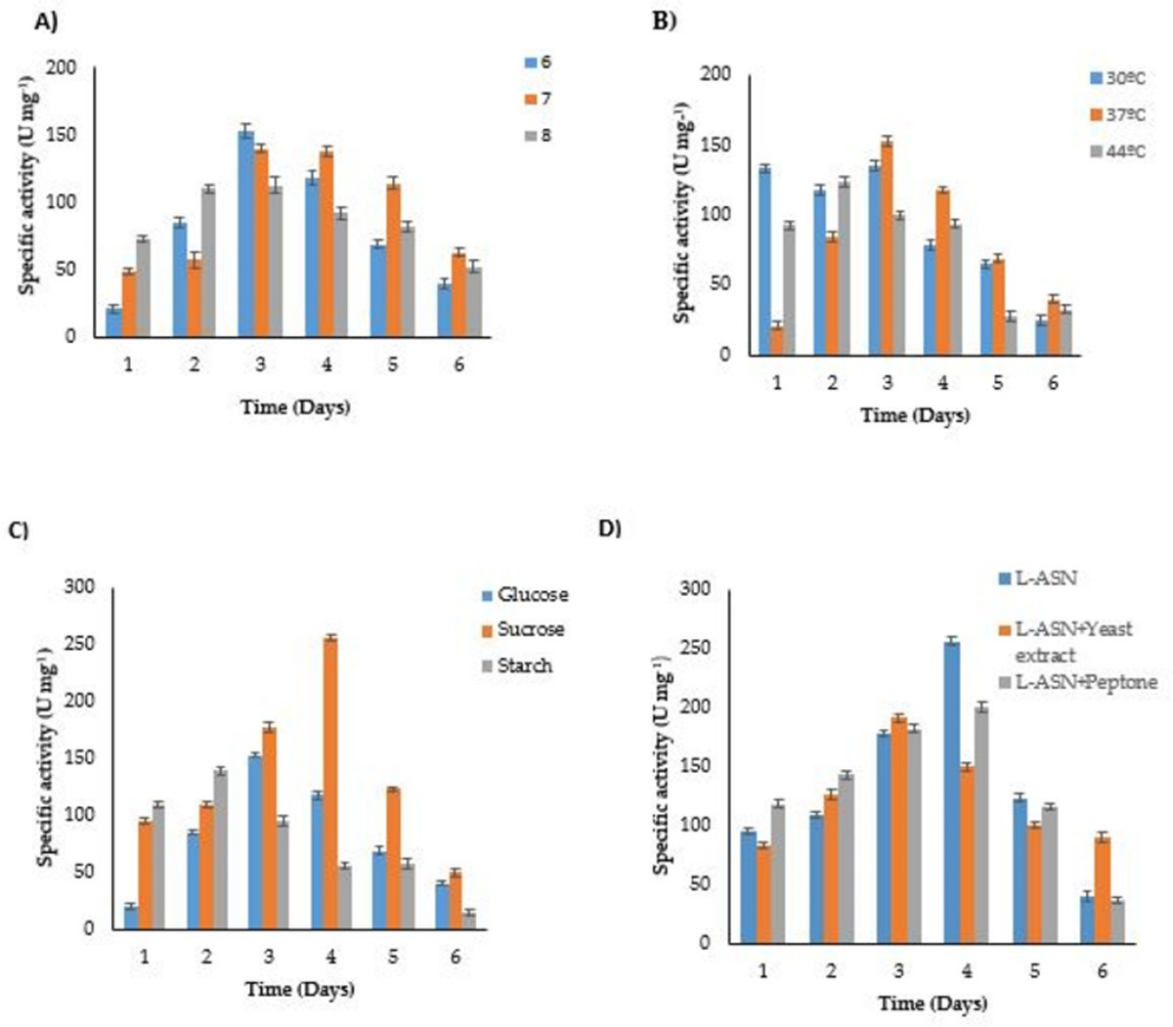
Effect of **A)** pH at 37°C **B)** temperature at pH 6.0. **C)** carbon source at pH 6 and 37°C **D)** nitrogen source at pH 6, sucrose (0.2%) and temperature 37°C in M9 medium on L-ASNase production from *B*. *halotolerans* ASN9.

L-asparaginase production was assessed at different pHs (6, 7, 8) and temperatures (30, 37, 44°C). *B*. *halotolerans* ASN9 showed maximum enzyme activity of 153 ± 0.63 U mg^-1^ at pH 6 and 37°C on third day of incubation which reduced to 40 U mg^-1^ on the sixth day of incubation. Enzyme production was comparatively low in pH 7 and 8 (Fig 1A). Similarly, temperatures higher or lower than 37°C resulted in decreased enzyme production (Fig 1B). Three carbon sources (glucose, sucrose and starch) were examined for optimum L-ASNase production. The bacterium showed maximum enzyme activity (256 U mg^-1^) on fourth day when grown in M9 medium with 0.2% sucrose and 1% L-asparagine. Slightly less L-ASNase was produced by ASN9 when either glucose (153 ±0.63 U mg^-1^) or starch (139±1.5 U mg^-1^) was used as a carbon source (Fig 1C). Optimization of nitrogen sources for L-ASNase production showed that the combination of L-asparagine with yeast extract (191±0.63 U mg^-1^) and peptone (200±0.74 U mg^-1^) decreased the enzyme specific activity (Fig 1D). These results collectively indicated that the strain under study has different and cheaper growth requirements to produce a handsome amount of extracellular L-ASNase, which establishes its biotechnological significance.

The results of the response surface model, including observed response values, are given in S2 Table. The analysis of the variance (ANOVA) on the data obtained after performing the RSM experimental design is given in S3 Table. The p-value was 0.0491, suggesting that the experimental model was significant in enhancing the L-ASNase production. Moreover, the R^2^ value (correlation coefficient) was 0.86 proposing a good regression fit of the experimental model. Additionally, the R^2^ adjusted value showed the correction decision coefficient was 0.73. The results and response of the model suggested a good fit model (S3 Table). Change in temperature and pH affects the L-ASNase production; as shown in model at temperature of 44°C low enzyme production was predicted at pH 6 and 7 (Run 10, Run 17) (S2 Table). p-values less than 0.0500 indicate model terms are significant. In this case A (pH) and B (temperature) are significant model terms (S3 Table). Three-dimensional response surface graphs were generated to recognize the relationship of pH, temperature and incubation time on L-ASNase (S3A and S3B Fig). According to the applied model, the predicted maximum value of *Bacillus halotolerans* ASN9 L-asparaginase was 143.8 U mg^-1^ and it can be obtained at temperature of 37°C, pH of 6, and at 3^rd^ day of incubation time (Run 2). The RSM model validates the experimental results. In experimental design maximum L-ASNase production was obtained within suggested parameters (pH, temperature and incubation time) of RSM that validates the significance of RSM model in predicting the optimized conditions for L-ASNase production.

### Purification of L-asparaginase

Cell free culture supernatant processed with 60% ice-cold acetone precipitated the protein with L-ASNase specific activity of 2360 U mg^-1^ protein quantity of 0.024 mg mL^-1^. L-asparaginase was purified up to 9.2-fold through acetone precipitation method with a yield of 48.9%. Precipitated L-ASNase was further purified through Sephadex G-100 column. Highest enzyme activity (370 U mL^-1^) was calculated in 12^th^ fraction of column chromatography (S4A Fig), with total enzyme activity of 1110 U, protein amount of 0.36 mg, and 12-fold purification (Table 2). The molecular weight of the purified L-ASNase was a monomer of 60 kDa (S4B Fig).

**Table 2:** Purification scheme for L-asparaginase of *B*. *halotolerans* ASN9.

*B*. *halotolerans* ASN9	Total Volume (mL)	Total Enzyme activity (U)	Total protein (mg)	Specific activity (U mg^-1^)	Fold purification	% Yield
**Crude**	500	4625	18	256	1	100
**Acetone precipitation**	40	2266	0.96	2360	9.2	48.9
**Sephadex column fraction (12)**	3	1110	0.36	3083	12	24

### Characterization of purified glutaminase free L-asparaginase from *B*. *halotolerans* ASN9

#### Effect of different pH and temperatures on L-asparaginase activity

**Fig 2:**
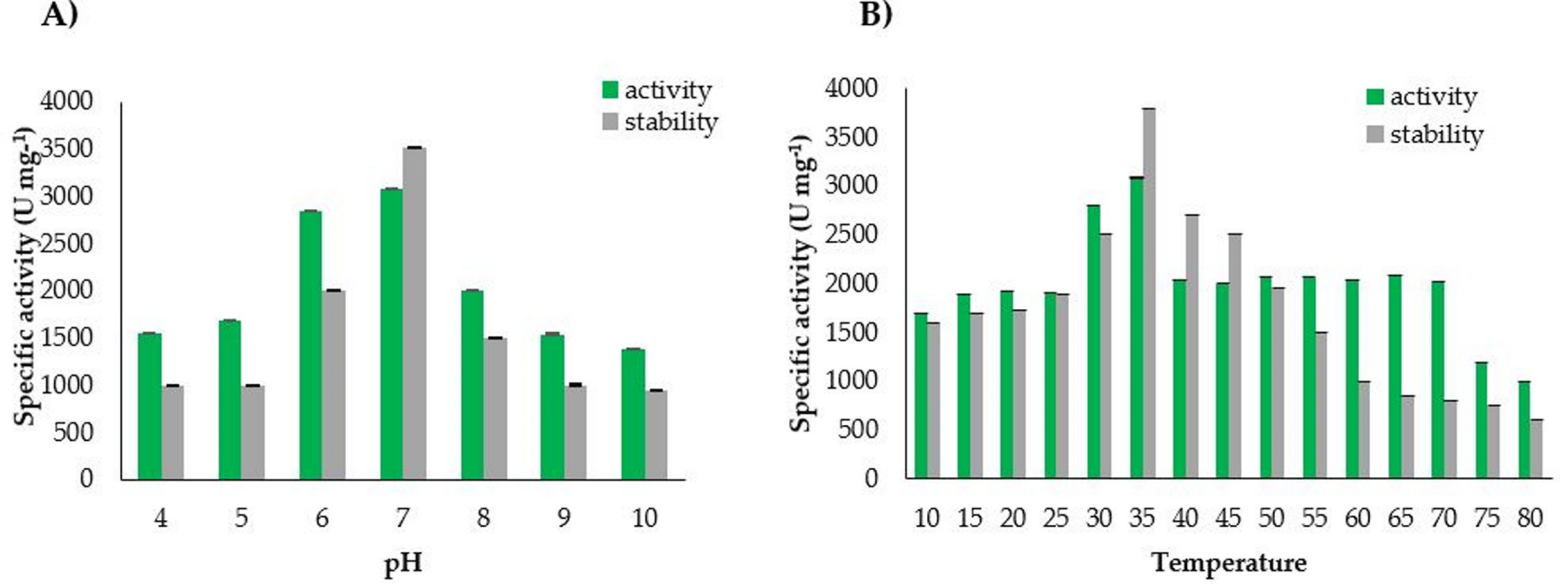
Effect of A) pH at 37°C B) temperature at pH 7 on the activity and stability of purified L-ASNase from *Bacillus halotolerans* ASN9 (bars represent the standard error of mean).

Purified L-ASNase retained its activity over a wide range of pH (4–10) while maximum activity (3083 U mg^-1^) and stability (3509 U mg^-1^) for the purified L-ASNase was recorded at neutral pH (7). Further increase in pH decreased the activity and stability of purified enzyme, with lowest specific activity of 1380 U mg^-1^ recorded at pH 10 (Fig 2A). Similarly, L-ASNase showed maximum specific activity (3083 U mg^-1^) and stability (3789 U mg^-1^) at 35°C temperature (Fig 2B). Overall, the enzyme showed its specific activity from 10 to 80°C in the range of 1000–3083 U mg^-1^ (Fig 2B). Higher specific activities of purified enzyme at physiological pH and temperature makes ASN9 strain a useful option for anticancer activity. L-ASNase retained 51% stability till 16 h at 37°C and pH 7 (S4C Fig).

#### Effect of surfactants, organic solvents, inhibitors, sodium chloride and metallic salts on the activity of purified L-asparaginase

**Fig 3:**
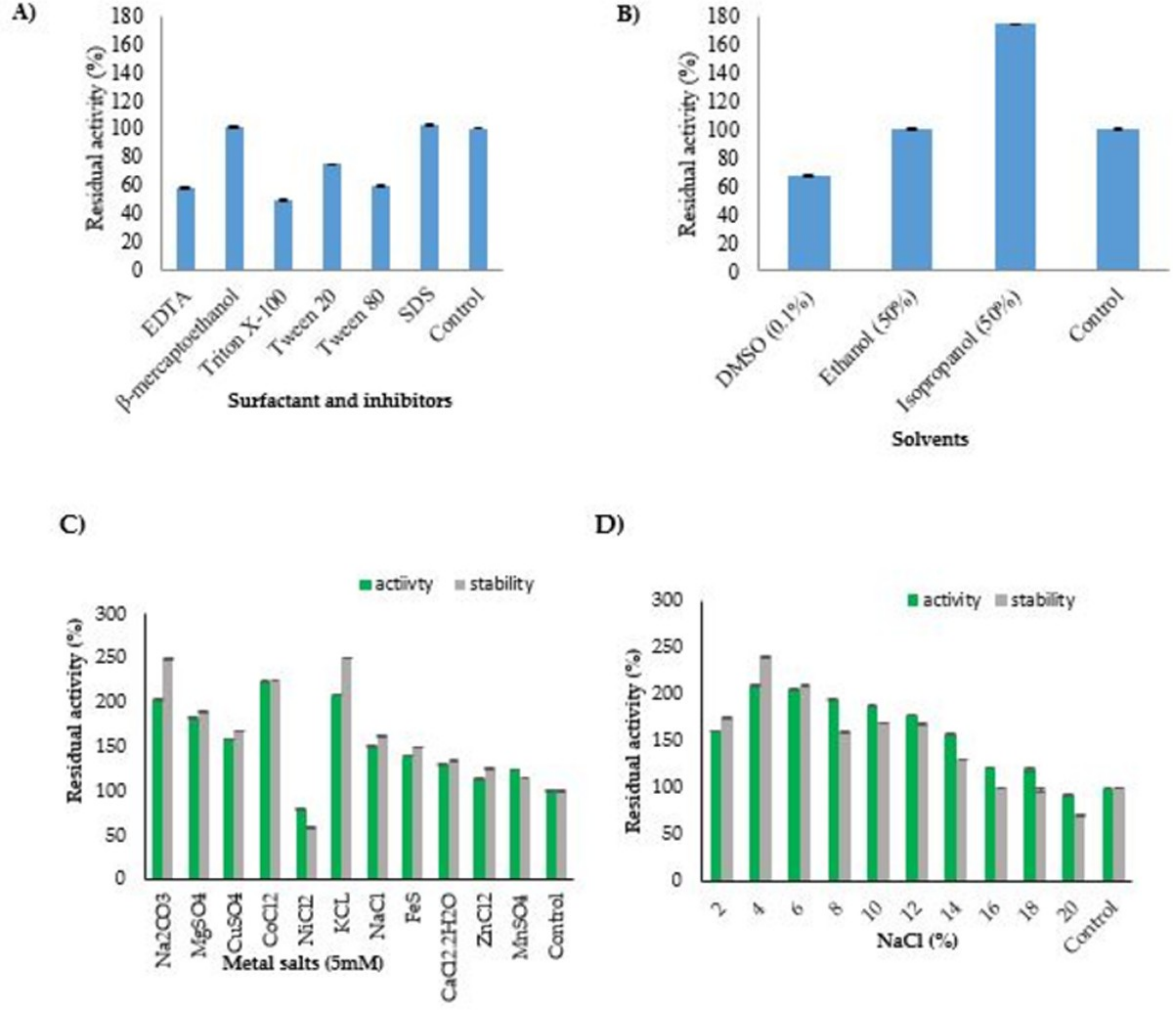
Effect of A) surfactants and inhibitors (1mM) B) solvents C) salts of mono- and di-valent metal ion (5 mM) D) NaCl concentration on the activity and stability of purified L-ASNase at 37°C and pH 7.

The impact of effectors on the enzyme activity was determined in terms of residual activity, which is the percentage of enzyme activity in the presence of effectors to that of untreated one. Enzyme treated with tween 80 and tween 20 lost 41% and 25.5% of activity, respectively. Triton-X 100 severely affected the activity of enzyme and drastically decreased the residual activity of the enzyme to 49%. EDTA also exhibited an inhibitory effect on enzyme activity and reduced the residual activity to 57% (Fig 3A). β-mercaptoethanol did not affect while SDS slightly enhanced the enzyme activity (Fig 3A). Purified L-ASNase retained its activity when treated with ethanol. Isopropanol enhanced the enzyme activity with a drastic increase in the residual activity to 175% (Fig 3B). When tested against different metal salts, the residual activity of purified L-ASNase increased. Only nickel salt inhibited the enzyme activity (residual activity 80%). On the other hand, cobalt enhanced the enzyme activity to about two folds with residual activity to 223% (Fig 3C). Increase in the ASN9 L-ASNase activity (upto 131%) was observed in the presence of calcium salt. L-asparaginase when treated with 4% NaCl improved the specific activity to 6,492 U mg^-1^, a slight decrease in specific activity (4,880 U mg^-1^) was observed after increasing salt concentration upto 14%. Further increase in the salt concentration (20% NaCl) led to a successive decrease in specific activity (2,843 U mg^-1^). Specific activity (2,098 U mg^-1^) of untreated L-ASNase was low in comparison to 2–18% NaCl treated L-ASNase (Fig 3D). Retention of activity in the presence of surfactants, organic solvents, inhibitors, sodium chloride and metal salts showed its potential to work in industrial processes.

#### Substrate specificity and kinetics of glutaminase free L-asparaginase

**Fig 4:**
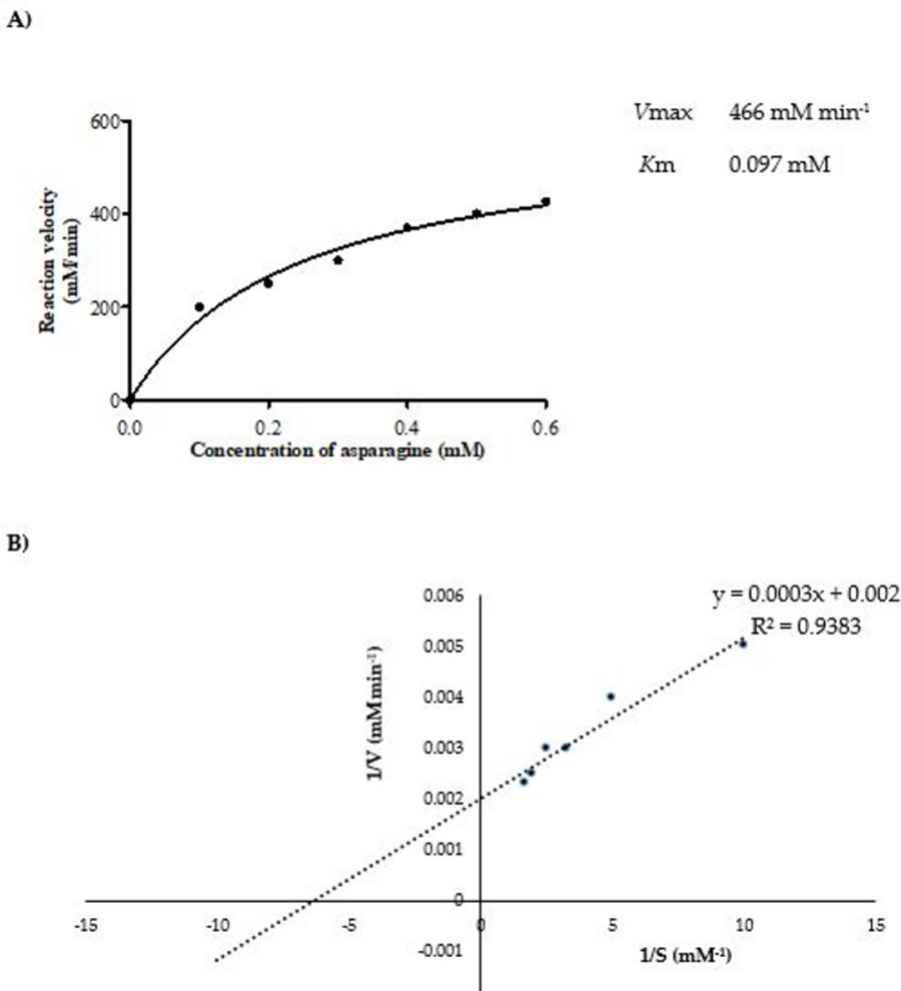
Determination of Kinetic parameters of purified L-ASNase from *B*. *halotolerans* ASN9 at 37°C and pH 7 by **A)** Michaelis-Menten plot (*K*m = 0.097 mM, *V*max = 466 mM min^-1^ **B)** Lineweaver-Burk plot (*K*m = 0.14, *V*max = 467 mM min^-1^).

Purified enzyme showed highest activity (3654±1.3 U mg^-1^) against 0.6 mM L-asparagine, while no enzyme activity against glutamine and urea was observed when treated with different concentrations of substrates (Table 3). *B*. *halotolerans* ASN9 L-ASNase reported here showed no activity for L-glutamine. Kinetics of L-ASNase showed a gradual increase in enzyme activity with increase in L-asparagine concentration from 0.1 to 0.6 mM. Michaelis-Menten curve was obtained for the initial velocity of reaction and different concentrations of the substrate using GraphPad Prism5. Calculated *K*m and *V*max values were 0.097± 0.01 mM and 466.4 mM min^-1^, respectively with R² value of 0.92 (best fit model) (Fig 4A). Calculated values of *K*m and *V*max were 0.14 mM and 467 mM min^-1^, respectively with R² value of 0.93 (best fit model) (Fig 4B).

**Table 3:** Substrate specificity of purified L-ASNase from *B*. *halotolerans* ASN9 at 37°C and pH 7.

Substrate	Concentration (mM)		
	0.2	0.4	0.6
**L-asparagine**	2576±1.5 (U mg^-1^)	3083±0.9 (U mg^-1^)	3654±1.3 (U mg^-1^)
**L-glutamine**	-	-	-
**Urea**	-	-	-

- no activity

#### Assessment of antioxidant and anticancer activity

Antioxidant activity of purified bacterial L-ASNase showed that the scavenging efficiency of L-ASNase on DPPH was enhanced by increasing the concentration of enzyme from 20 to 120 μg mL^-1^. The inhibitory activity of DPPH radicals was calculated in percentage and ranged between 35 to 62% with **IC**_50_ of 70.7 μg mL^-1^ (S4 Table). This indicated the potential of purified glutaminase free L-ASNase to act as an antioxidant agent.

**Fig 5:**
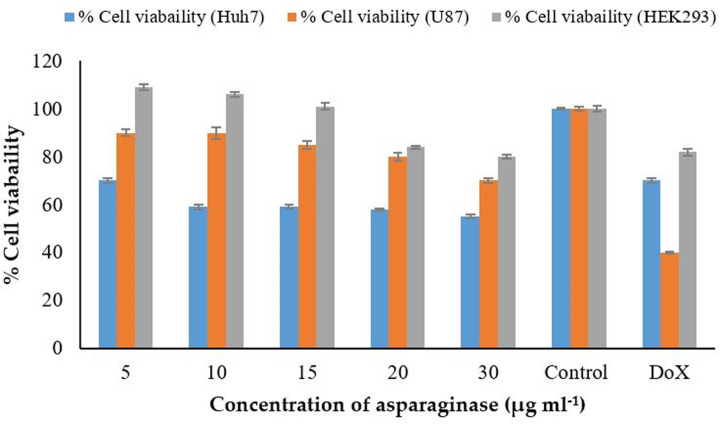
Cell viability of Huh7 and U87 after treatment with purified L-ASNase. Narrow bars represent the standard error of mean. Control presents the cells grown without L-ASNase and DoX represents the standard drug Doxorubicin treated cells at concentration of 0.18 μM.

The effect of purified L-ASNase on U87 (human malignant glioma) and Huh7 (hepatocellular carcinoma) cell viability was evaluated in a dose-dependent manner by MTT assay (5–30μg mL^-1^). Notable antiproliferative activity for U87 was observed with **IC**_50_ value of 55 μg mL^-1^ of purified L-ASNase. Relatively low **IC**_50_ of 37 μg mL^-1^ was observed for the antiproliferative activity of Huh7 hepatocellular carcinoma cell lines. The percentage viability of U87 and Huh7 was 40 and 70%, respectively in the presence of reference drug doxorubicin (0.18 μM) (Fig 5). Percentage viability of U87 and Huh7 was 70 and 55%, respectively when treated with 30 μg mL^-1^ of purified L-ASNase from ASN9. Anticancer efficiency of purified L-ASNase from *B*. *halotolerans* ASN9 against Huh7 was greater than the standard drug DoX (Fig 5). It is the first report of anticancer activity of L-ASNase from *B*. *halotolerans* against U87 and Huh7 (S5A-S5D Fig). Percentage cell viability remained greater than 80% at concentrations between 5–30 μg mL^-1^ of the purified L-ASNase at non-cancerous HEK293 cell line (S5E Fig). Our results thus indicated that purified glutaminase free L-ASNase from *B*. *halotolerans* ASN9 may be a promising anticancer enzyme for various cancers.

## Discussion

The current study is based on the production, purification, and characterization of asparaginase from *B*. *halotolerans* ASN9. Bacterial asparaginases are preferred over others on the basis of biotechnological conveniences. Commercially available formulations of *E*. *coli* and *Erwinia chrysanthemi* asparaginases that are considered best for clinical applications have shown the cross reactivity with glutamine and urea [23]. Hence exploration of new bacterial sources of asparaginase is always in demand. Present study is focused on the production, purification and characterization of L-ASNase from indigenous *B*. *halotolerans* ASN9. Screening of L-ASNase producing bacteria was carried out on M9 minimal medium supplemented with phenol red. El-Fakharany and his coworkers (2020) isolated asparaginase producing *B*. *halotolerans* OHEM18 from Egyptian soil samples [21]. The strain was identified on Czapek-Dox agar supplemented with phenol red with a prevalent pink zone surrounding the growth of strain, an approach similar to what was employed in this study [23].

Optimization experiments revealed that *B*. *halotolerans* ASN9 produced maximum enzyme (265 U mL^-1^) under submerged growth on the third day of incubation, at 37°C, when acidic pH of 6 was maintained in M9 medium supplemented with sucrose (0.2%) as carbon source and L-asparagine (1%) as nitrogen source. RSM is a powerful tool for identifying and optimizing the optimum factors for enhancing enzyme production. Statistical optimization of pH, temperature and incubation time for production medium using RSM demonstrated that the pH and temperature have significant effect on activity. RSM and experimental results revealed pH 6, temperature 37°C at third day of incubation as optimum condition for *B*. *halotolerans* ASN9 L-ASNase production. The pH and temperature of growth medium affects the enzyme production by differing the movement of components from cell membrane [24]. Similar pH of 6.5 was reported for optimum production of L-ASNase from *B*. *licheniformis* and *B*. *subtilis* [25–27]. Nutritional requirements also play a vital role for maximal production of L-ASNase and it varies among microorganism. Glucose act as best carbon source for L-ASNase production in cells of *B*. *licheniformis* [27]. Optimized production of asparaginase (37.93 IU mL^-1^) from *Bacillus australimaris* was also reported in M9 medium supplemented with 2.5% asparagine [28]. Vimal et al. used 0.1% lactose and 0.2% asparagine along with the combination of tryptone and yeast extract to produce 3.82 IU mL^-1^ of asparaginase from *Escherichia coli* K-12 [29]. Our results are in contrast with the previously reported optimized ingredients of submerged medium. These results collectively indicate that strain under study has different growth requirements that favor their optimal asparaginase production. Strain ASN9 has simpler and cheaper growth requirements and produces handsome amounts of extracellular asparaginase, which establishes its biotechnological significance.

Further asparaginase was precipitated and partially purified using 60% of ice chilled acetone. The resulting preparation produced 270 U mL^-1^ of enzyme activity, around 1.2 mg/mL of protein and 225 U mg^-1^ specific activity. Partially purified asparaginase was further purified through Sephadex G-100 column. This purification enhanced the enzyme activity to 370 U mL^-1^ and specific activity to 3173 U mg^-1^, with 5.14-fold purification and 0.42% recovered yield. In contrast to our results, previously reported *B*. *halotolerans* OHEM18 asparaginase was partially purified through 65% ammonium sulfate precipitation method with specific activity of 55.97 U mg^-1^ and 2.2 fold purification [21]. Whereas El-Fakharany purified the enzyme through anion exchange chromatography QFF column with 3.84 fold purification and specific activity of 123.3 U mg^-1^ [21]. Specific activity of presently reported purified asparaginase of *B*. *halotolerans* ASN9 is greater than the previously reported OHEM18 strain. The molecular weight of the purified asparaginase from ASN9 was found to be 60 kDa. Whereas, asparaginase from *E*. *chrysanthemi* was of 138 kDa, and asparaginase from *B*. *subtilis* was of 40 kDa [26, 30, 31].

The purified *B*. *halotolerans* ASN9 asparaginase functions optimally around pH 7, which is near to pH of human blood. Moreover, optimum activity of asparaginase was observed near 37°C, which is also the physiological temperature of human body. Interestingly, the purified asparaginase showed activity even at a higher temperature of 70°C with high specific activity (2220 U/mg). The optimum temperature and pH of *B*. *halotolerans* ASN9 is similar to the reported bacterial asparaginases (S5 Table) [3, 32]. Higher specific activities of purified enzyme at physiological pH and temperature makes current ASN9 strain a useful option for anticancer activity. Similarly, retention of enzyme activity at high temperature favors its application in acrylamide reduction in food industry. Enzyme kinetics showed *K*m value of 0.097 mM for *B*. *halotolerans* ASN9 asparaginase, which is comparable to the currently used *E*. *coli* and *Erwinia* asparaginase with high specificity of 3173.07 U/mg (S5 Table) [33–37]. However, most of the previously reported L-asparaginases showed specific activity for L-glutamine and urea, with values corresponding to a range of 2–10% for their L-asparaginase activities [19, 21]. *B*. *halotolerans* ASN9 asparaginase reported here showed no specificity for L-glutamine and hence its cross-reactivity problems will be minimal in pharmaceutical industry.

*B*. *halotolerans* ASN9 asparaginase activity increased in the presence of metallic salts of sodium, magnesium, copper, cobalt, potassium, iron, calcium, zinc and manganese. Our results contrast with El-Fakharany study where all metallic ions (Ca^2+,^ Hg^2+^ and Cu^2+^, Mg^2+^, K^+^ and Zn^2+^ decreased the enzyme activity [21]. Metallic ions always play a vital role in thermal stability and enzyme activity [22]. ASN9 asparaginase activity increased upto 131% in the presence of calcium salt. Calcium regulates the killing effect of cancer cells by natural killer cells and T-lymphocytes. Increased asparaginase activity in presence of calcium ions strongly favors the use of current enzyme in cancer therapy, as the cell proliferation and apoptosis of cancer cells is dependent on intracellular calcium concentration [38].

Antioxidant potential of purified asparaginase from ASN9 was investigated using DPPH radical scavenging assay. Inhibition activity of DPPH radicals was ranged from 35.5±0.43 to 62.5±1.02% (in 20–120 μg mL^-1^ of L-ASNase) with **IC**_50_ value of 70.7 μg mL^-1^. These results are in agreement with previously reported L-ASNase from *B*. *halotolerans* OHEM18 having antioxidant activity of 13.6–93.3% (in 15–480 μg mL^-1^ of L-ASNase) [21]. Antioxidant activity of this enzyme makes it a potential candidate for elimination and neutralization of oxidative stress in cells.

Considering as chemotherapeutic candidate; U87 glioma and Huh7 hepatocellular carcinoma cells were treated with purified asparaginase of *B*. *halotolerans* ASN9 and significant anticancer activity with **IC**_50_ of 55 and 37 μg mL^-1^ were obtained, respectively. Anticancer activity (45%) of purified asparaginase (30 μg mL^-1^) on Huh7 is higher than the anticancer activity (30%) of DoX. It is the first report of anticancer activity of asparaginase from *B*. *halotolerans* against U87 and Huh7. Moreover, the results of the cytotoxicity assay suggest that *B*. *halotolerans* ASN9 L-ASNase’s antiproliferative activity seems to be selective for cancer cells as much less toxicity against normal cells (HEK293) was observed even at comparatively higher concentrations (30 μg mL^-1^). Moharib, (2018) reported that asparaginase from *Vigna unguiculata* killed 50% of HELLA, HCT116 and HEPG2 cancer cell lines [38]. Asparaginase from *Capsicum annum* L. which had anticancer activity against three different cell lines KB, A549 and HELLA [39]. El-Fakharany reported the cell cycle distribution of NFS60 cells when treated with 10, 15 and 20 mg mL^-1^ of L-asparaginase [21].

## Conclusion

In this research L-ASNase from *Bacillus halotolerans* ASN9 was purified and characterized for robustness and commercial applications. The extracellular L-ASNase from *Bacillus halotolerans* ASN9 was found to have higher specificity for asparagine with better biochemical properties and specific enzyme activity in comparison to reported strains. Retention and improvement of activity in the presence of a variety of protein inhibitors, mono and divalent metallic salts evidenced its commercial importance. Further studies on protein and genetic engineering of current strain could improve the anticancer potential of asparaginase for industrial application.

## Supporting information

S1 Table Sample conditions and screening of asparaginase producing bacterial isolates from soil/water samples through hydrolytic zone formed on M9 medium containing 1% ASN. (PDF)

S2 Table Box–Behnken matrix for analysis of parameters: pH, temperature and incubation time for optimization of the L-ASNase by *B*. *halotolerans* ASN9. (PDF)

S3 Table ANOVA for L-ASNase production, adjusted from experimental results obtained with Box–Behnken plot. (PDF)

S4 Table DPPH free radical scavenging activities of L-asparaginase. (PDF)

S5 Table Summary of biochemical/kinetics of different L-asparaginases from different bacterial sources. (PDF)

S1 Fig **A.** Qualitative screening of asparaginase producing bacteria. **B.** Asparaginase production assay of five distinct isolates grown in M9 medium at 37°C and pH 6.0. Results represent a mean of three experimental replicates and error bars represent the standard error of mean.
(ZIP)

S2 Fig Phylogenetic analysis of *B*. *halotolerans* ASN9 16S rRNA gene with closely related *Bacillus* species using neighbor-joining method. Accession numbers and % similarity is indicated for each entry. Branch points show bootstrap percentages (1000 replicates).
(TIF)

S3 Fig **A.** Three-dimensional response surface plots showing the interactive effects of pH and incubation time on L-ASNase activity. **B.** Three-dimensional response surface plots showing the interactive effects of temperature and incubation time on L-ASNase activity.
(ZIP)

S4 Fig **A.** Elution profile for Sephadex G-100 column chromatography equilibrated with Tri-HCl buffer of pH 7.4. Elution flow rate was 3.0 ml/fraction and absorbance at 280 nm was recorded. Fraction 12 showed high asparaginase activity. **B.** SDS-PAGE of L-asparaginase **Lane 1.** Protein marker **Lane 2.** Acetone precipitated proteins **Lane 3.** Purified L-ASNase after Sephadex G-100 column chromatography. **C.** Effect of incubation time on stability of L-ASNase at pH 7 and 37°C (bars represent the standard error of the mean).
(ZIP)

S5 Fig **A.** Anticancer activity (30%) of purified asparaginase at concentration of 30 μg ml-1 on U87 human malignant glioma cells. **B.** Untreated U87 human malignant glioma cells. **C.** Anticancer activity (55%) of purified L-asparaginase at concentration of 30 μg ml-1 on Huh7 hepatocyte carcinoma cells. **D.** Untreated Huh7 hepatocyte carcinoma cells. **E.** Cell viability (80%) of HEK293 human embryonic kidney cells with purified L-asparaginase at concentration of 30 μg ml-1. **F.** Untreated HEK293 human embryonic kidney.
(ZIP)
