# Supplementary material for: Characterization and applications of glutaminase free L-asparaginase from indigenous Bacillus halotolerans ASN9
Source: PLoS One. 2023 Nov 28;18(11):e0288620. doi: 10.1371/journal.pone.0288620 (PMC10683992; doi:10.1371/journal.pone.0288620)
Supplement: S1 Table — (PDF) [file pone.0288620.s001.pdf]

S1 Table. Sample conditions and screening of asparaginase producing bacterial isolates from soil/water samples through hydrolytic zone formed on M9 medium containing 1% ASN.

**Isolates**	**Source**	**Temperature of sample**	**pH of sample**	**Zone of hydrolysis (mm) Mean ± SE**
ASN4	Garden soil (Islamabad, Pakistan)	8	8.5	8±0.7
ASN9	Garden soil (Islamabad, Pakistan)	8	8.5	19±0.2
ASN12	Garden soil (Okara, Pakistan)	10	8.6	11±0.1
ASN14	Agriculture soil from wheat field (Jaranwala, Pakistan)	10	8.6	11±0.7
ASN38	Water sample (Islamabad, Pakistan)	16	7.5	10±0.8

(SE presents standard error of mean)
