# Supplementary material for: Characterization and applications of glutaminase free L-asparaginase from indigenous Bacillus halotolerans ASN9
Source: PLoS One. 2023 Nov 28;18(11):e0288620. doi: 10.1371/journal.pone.0288620 (PMC10683992; doi:10.1371/journal.pone.0288620)
Supplement: S2 Table — (PDF) [file pone.0288620.s002.pdf]

S2 Table. Box–Behnken matrix for analysis of parameters: pH, temperature and incubation time for optimization of the L-ASNase by *B. halotolerans* ASN9.

**Run**	**pH**	**Temperature °C**	**Incubation Time (Days)**	**Actual value U mg^{-1}**	**Predicted value U mg^{-1}**
1	7	37	3	140	139.44
2	6	37	3	153.00	143.88
3	6	37	1	21.00	45.30
4	7	37	3	140.00	139.44
5	7	37	3	140.00	139.44
6	6	37	3	153.00	132.28
7	8	37	3	113.00	135.13
8	8	37	3	113.00	135.13
9	6	37	6	40.00	57.14
10	6	44	3	100.00	100.00
11	8	37	1	72.00	44.88
12	7	37	1	48.00	50.82
13	7	37	3	140.00	139.44
14	8	37	6	82.00	64.86
15	6	30	3	136.00	136.00
16	7	37	3	140.00	139.44
17	7	44	1	93.00	93.00
