# Supplementary material for: Characterization and applications of glutaminase free L-asparaginase from indigenous Bacillus halotolerans ASN9
Source: PLoS One. 2023 Nov 28;18(11):e0288620. doi: 10.1371/journal.pone.0288620 (PMC10683992; doi:10.1371/journal.pone.0288620)
Supplement: S3 Table — (PDF) [file pone.0288620.s003.pdf]

S3 Table. ANOVA for L-ASNase production, adjusted from experimental results obtained with Box–Behnken plot.

Source	Sum of Squares	df	Mean Square	F-value	p-value	
**Model**	3300.63	8	412.58	3.46	0.0491	significant
**A-pH**	914.18	1	914.18	7.67	0.0243	
**B-Temperature**	72.56	1	72.56	0.6089	0.4577	
**C-Incubation time**	318.63	1	318.63	2.67	0.1407	
**AB**	325.43	1	325.43	2.73	0.1370	
**AC**	257.29	1	257.29	2.16	0.1799	
**A^2**	58.50	1	58.50	0.4909	0.5034	
**B^2**	761.49	1	761.49	6.39	0.0354	
**C^2**	526.32	1	526.32	4.42	0.0688	
**Residual**	953.37	8	119.17			
**R^2**	0.8659					
**R^2-adj**	0.7319					

df, indicates for degree of freedom.
