# Supplementary material for: Characterization and applications of glutaminase free L-asparaginase from indigenous Bacillus halotolerans ASN9
Source: PLoS One. 2023 Nov 28;18(11):e0288620. doi: 10.1371/journal.pone.0288620 (PMC10683992; doi:10.1371/journal.pone.0288620)
Supplement: S4 Table — (PDF) [file pone.0288620.s004.pdf]

S4 Table. DPPH free radical scavenging activities of L-asparaginase

Concentration ($\mu g\ ml^{-1}$)	Scavenging activity (%)
20	35.5 ± 0.43
40	45.4 ± 0.82
60	50.8 ± 0.81
80	51.7 ± 1.06
100	53.5 ± 0.72
120	62.5 ± 1.02
**IC_{50}**	70.7 $\mu g\ ml^{-1}$

IC_{50} is half-maximum inhibitory concentration

All values are expressed as mean ± SE
