# Supplementary material for: Characterization and applications of glutaminase free L-asparaginase from indigenous Bacillus halotolerans ASN9
Source: PLoS One. 2023 Nov 28;18(11):e0288620. doi: 10.1371/journal.pone.0288620 (PMC10683992; doi:10.1371/journal.pone.0288620)
Supplement: S5 Table — (PDF) [file pone.0288620.s005.pdf]

S5 Table. Summary of biochemical/kinetics of different L-asparaginases from different bacterial sources.

Source of L-asparaginase	Molecular size (kDa)	Optimum temperature (°C)	Optimum pH	Specific activity (U mg^{-1})	*K*m (mM)	References
Erwinia chrysanthemi	–	45	7.5	312.8	0.5	[3]
Erwinia carotovora	125–145	50	8.0	–	0.018	[34]
Cladosporium sp.	120	30	6.3	83.3	0.1	[35]
Bacillus licheniformis	134.8	40	9.0	697.09	0.014	[25]
Bacillus tequilensis	–	35	8.5	10.19	0.045	[36]
Bacillus subtilis	–	40	7.5	–	0.43	[26]
Vigna unguiculata	70	40	8.0	–	1.25	[32]
Pseudomonas fluorescens	141	34	6.3	0.94	109.9	[38]
Phaseolus vulgaris	79	37	8.0	846	6.72	[39]
Bacillus halotolerans OHEM18	41.5	40	8.2	215.33	4.7	[21]
Bacillus halotolerans ASN9	60	37	7	3173	0.0973	Current study
