# Supplementary figures and images for: Characterization and applications of glutaminase free L-asparaginase from indigenous Bacillus halotolerans ASN9
Source: PLoS One. 2023 Nov 28;18(11):e0288620. doi: 10.1371/journal.pone.0288620 (PMC10683992; doi:10.1371/journal.pone.0288620)

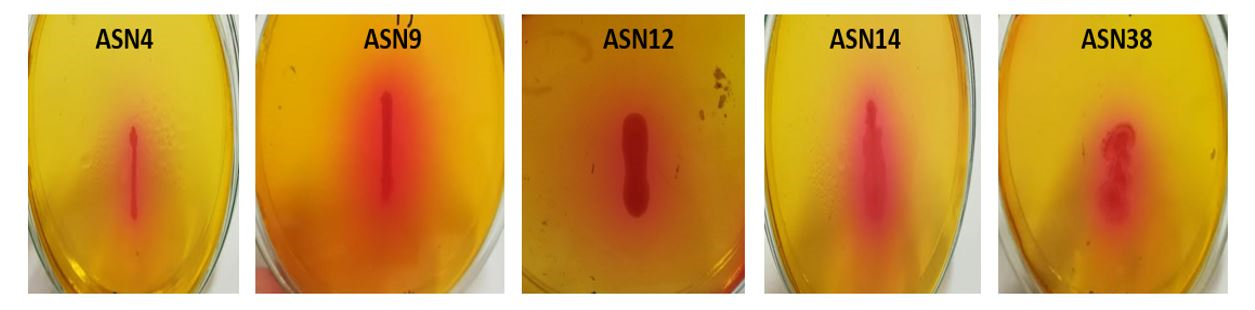

Supplement: S1 Fig — A. Qualitative screening of asparaginase producing bacteria. B. Asparaginase production assay of five distinct isolates grown in M9 medium at 37°C and pH 6.0. Results represent a mean of three experimental replicates and error bars represent the standard error of mean. (ZIP) [file pone.0288620.s006.zip › S1A_Fig.tif]

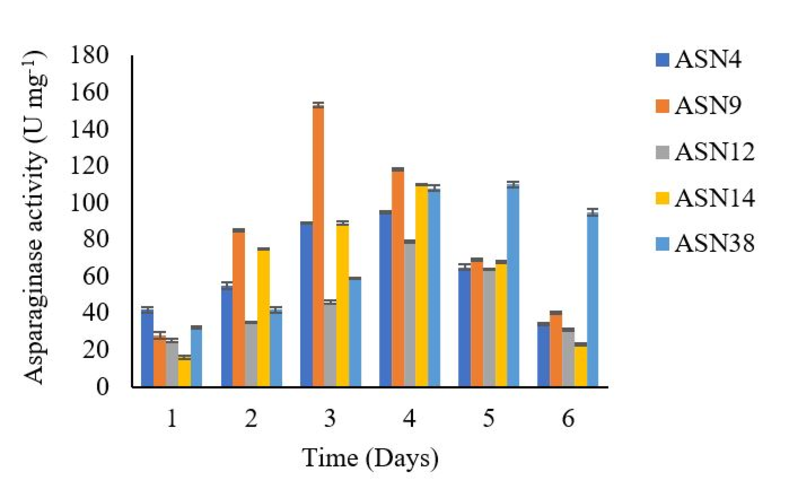

Supplement: S1 Fig — A. Qualitative screening of asparaginase producing bacteria. B. Asparaginase production assay of five distinct isolates grown in M9 medium at 37°C and pH 6.0. Results represent a mean of three experimental replicates and error bars represent the standard error of mean. (ZIP) [file pone.0288620.s006.zip › S1B_Fig.tif]

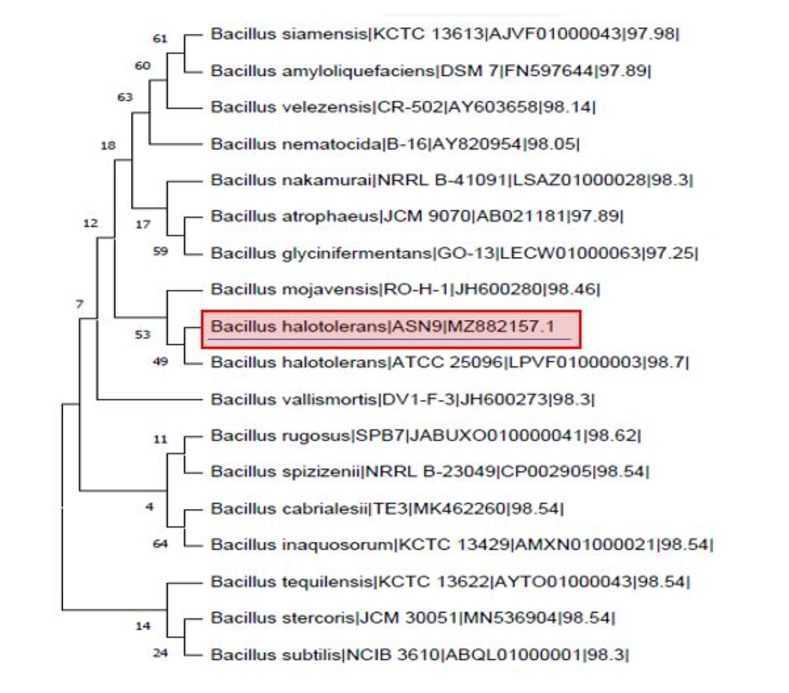

Supplement: S2 Fig — Accession numbers and % similarity is indicated for each entry. Branch points show bootstrap percentages (1000 replicates). (TIF) [file pone.0288620.s007.tif]

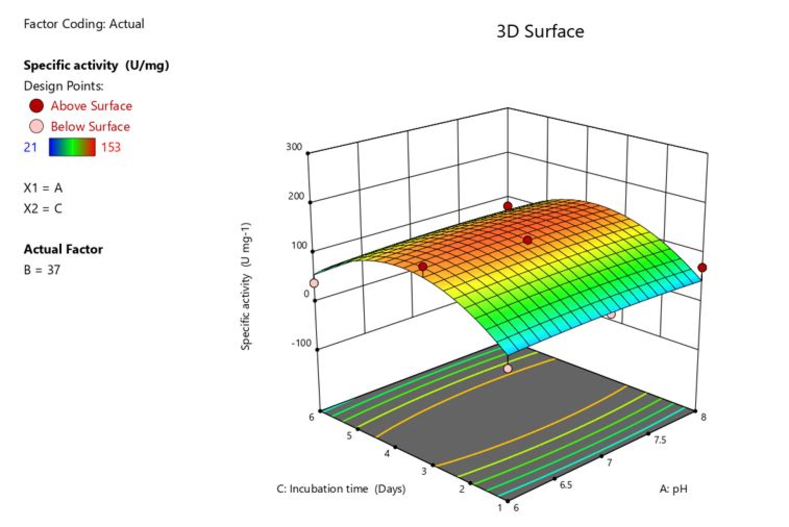

Supplement: S3 Fig — A. Three-dimensional response surface plots showing the interactive effects of pH and incubation time on L-ASNase activity. B. Three-dimensional response surface plots showing the interactive effects of temperature and incubation time on L-ASNase activity. (ZIP) [file pone.0288620.s008.zip › S3A_Fig.tif]

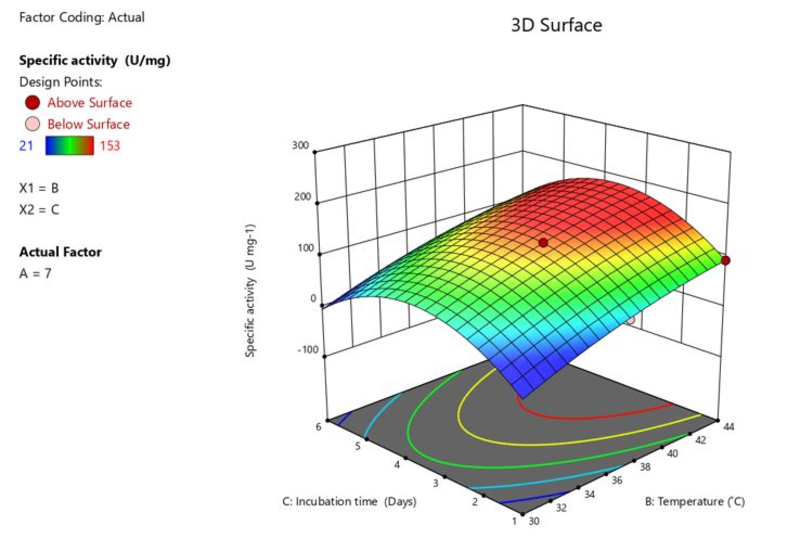

Supplement: S3 Fig — A. Three-dimensional response surface plots showing the interactive effects of pH and incubation time on L-ASNase activity. B. Three-dimensional response surface plots showing the interactive effects of temperature and incubation time on L-ASNase activity. (ZIP) [file pone.0288620.s008.zip › S3B_Fig.tif]

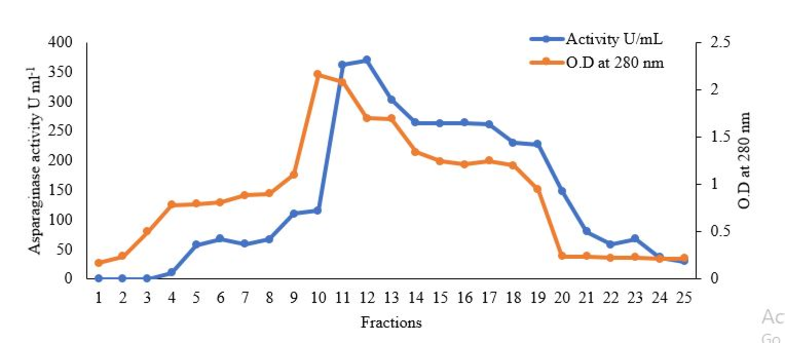

Supplement: S4 Fig — A. Elution profile for Sephadex G-100 column chromatography equilibrated with Tri-HCl buffer of pH 7.4. Elution flow rate was 3.0 ml/fraction and absorbance at 280 nm was recorded. Fraction 12 showed high asparaginase activity. B. SDS-PAGE of L-asparaginase Lane 1. Protein marker Lane 2. Acetone precipitated proteins Lane 3. Purified L-ASNase after Sephadex G-100 column chromatography. C. Effect of incubation time on stability of L-ASNase at pH 7 and 37°C (bars represent the standard error of the mean). (ZIP) [file pone.0288620.s009.zip › S4A_Fig.tif]

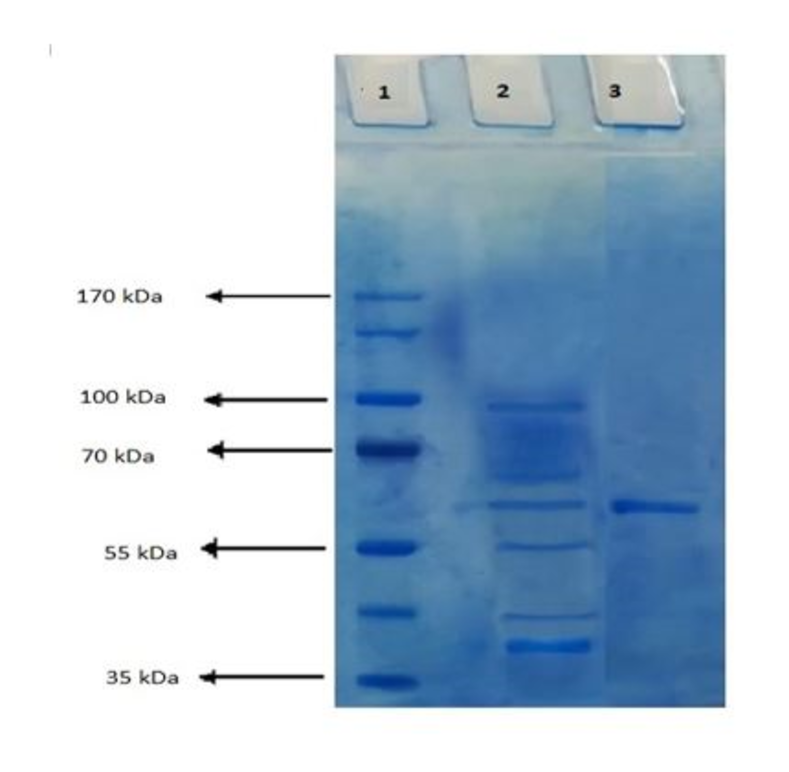

Supplement: S4 Fig — A. Elution profile for Sephadex G-100 column chromatography equilibrated with Tri-HCl buffer of pH 7.4. Elution flow rate was 3.0 ml/fraction and absorbance at 280 nm was recorded. Fraction 12 showed high asparaginase activity. B. SDS-PAGE of L-asparaginase Lane 1. Protein marker Lane 2. Acetone precipitated proteins Lane 3. Purified L-ASNase after Sephadex G-100 column chromatography. C. Effect of incubation time on stability of L-ASNase at pH 7 and 37°C (bars represent the standard error of the mean). (ZIP) [file pone.0288620.s009.zip › S4B_Fig.tif]

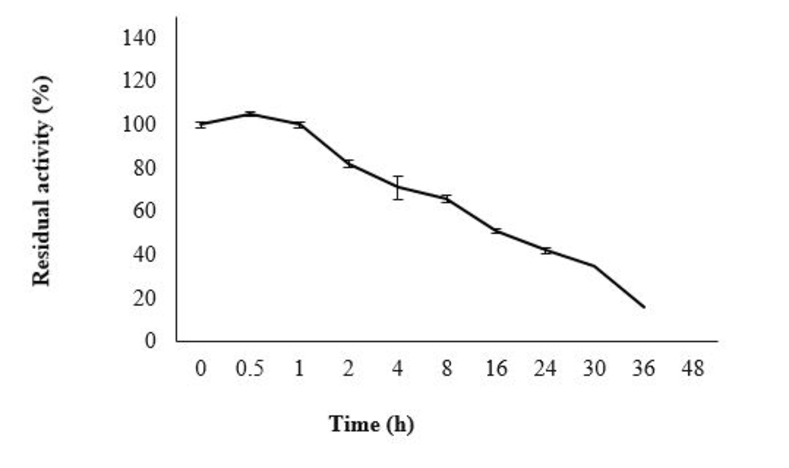

Supplement: S4 Fig — A. Elution profile for Sephadex G-100 column chromatography equilibrated with Tri-HCl buffer of pH 7.4. Elution flow rate was 3.0 ml/fraction and absorbance at 280 nm was recorded. Fraction 12 showed high asparaginase activity. B. SDS-PAGE of L-asparaginase Lane 1. Protein marker Lane 2. Acetone precipitated proteins Lane 3. Purified L-ASNase after Sephadex G-100 column chromatography. C. Effect of incubation time on stability of L-ASNase at pH 7 and 37°C (bars represent the standard error of the mean). (ZIP) [file pone.0288620.s009.zip › S4C_Fig.tif]

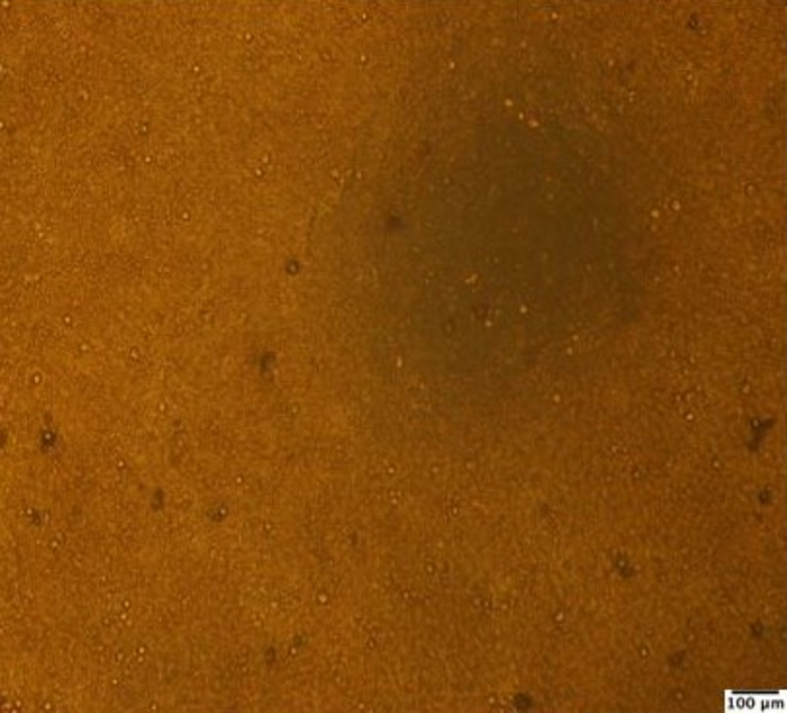

Supplement: S5 Fig — A. Anticancer activity (30%) of purified asparaginase at concentration of 30 μg ml-1 on U87 human malignant glioma cells. B. Untreated U87 human malignant glioma cells. C. Anticancer activity (55%) of purified L-asparaginase at concentration of 30 μg ml-1 on Huh7 hepatocyte carcinoma cells. D. Untreated Huh7 hepatocyte carcinoma cells. E. Cell viability (80%) of HEK293 human embryonic kidney cells with purified L-asparaginase at concentration of 30 μg ml-1. F. Untreated HEK293 human embryonic kidney. (ZIP) [file pone.0288620.s010.zip › S5A_Fig.tif]

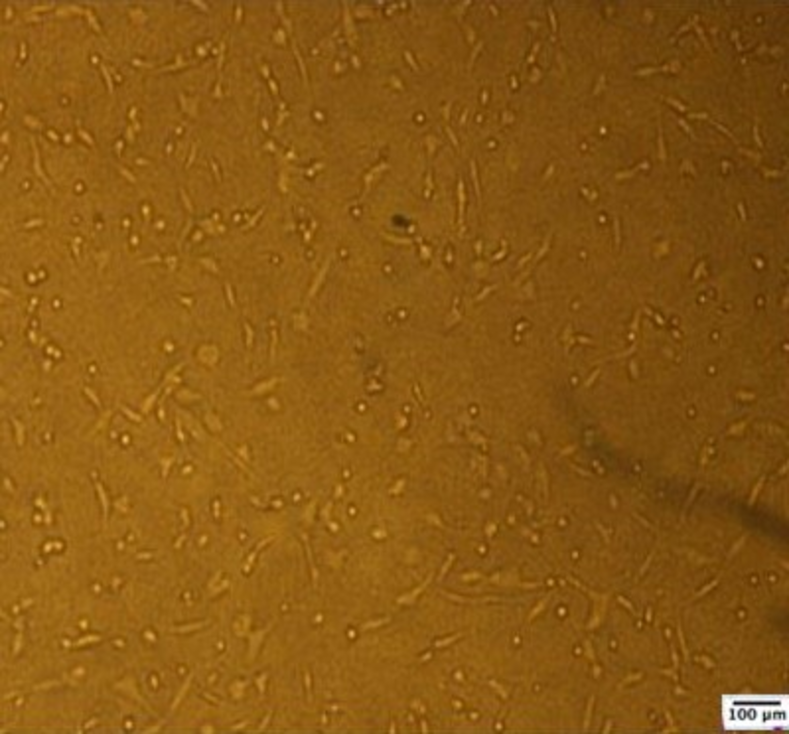

Supplement: S5 Fig — A. Anticancer activity (30%) of purified asparaginase at concentration of 30 μg ml-1 on U87 human malignant glioma cells. B. Untreated U87 human malignant glioma cells. C. Anticancer activity (55%) of purified L-asparaginase at concentration of 30 μg ml-1 on Huh7 hepatocyte carcinoma cells. D. Untreated Huh7 hepatocyte carcinoma cells. E. Cell viability (80%) of HEK293 human embryonic kidney cells with purified L-asparaginase at concentration of 30 μg ml-1. F. Untreated HEK293 human embryonic kidney. (ZIP) [file pone.0288620.s010.zip › S5B_Fig.tif]

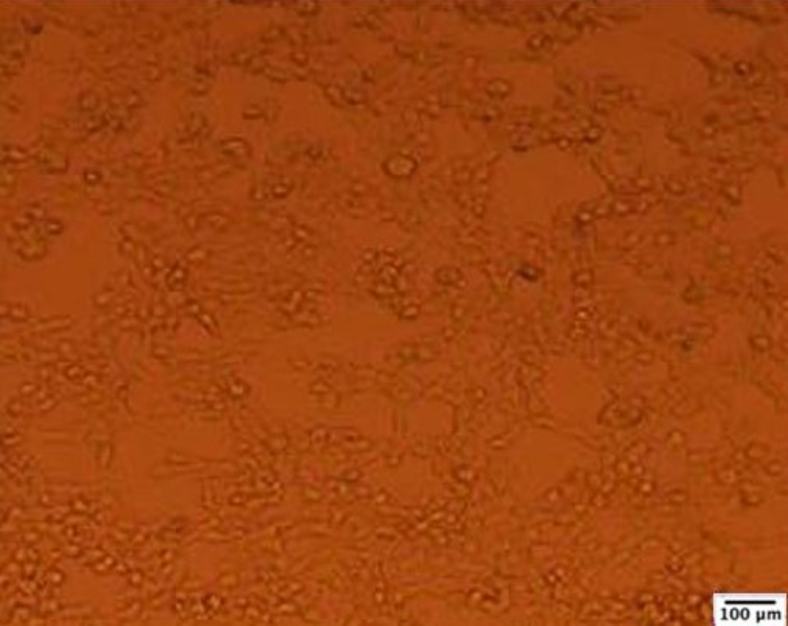

Supplement: S5 Fig — A. Anticancer activity (30%) of purified asparaginase at concentration of 30 μg ml-1 on U87 human malignant glioma cells. B. Untreated U87 human malignant glioma cells. C. Anticancer activity (55%) of purified L-asparaginase at concentration of 30 μg ml-1 on Huh7 hepatocyte carcinoma cells. D. Untreated Huh7 hepatocyte carcinoma cells. E. Cell viability (80%) of HEK293 human embryonic kidney cells with purified L-asparaginase at concentration of 30 μg ml-1. F. Untreated HEK293 human embryonic kidney. (ZIP) [file pone.0288620.s010.zip › S5C_Fig.tif]

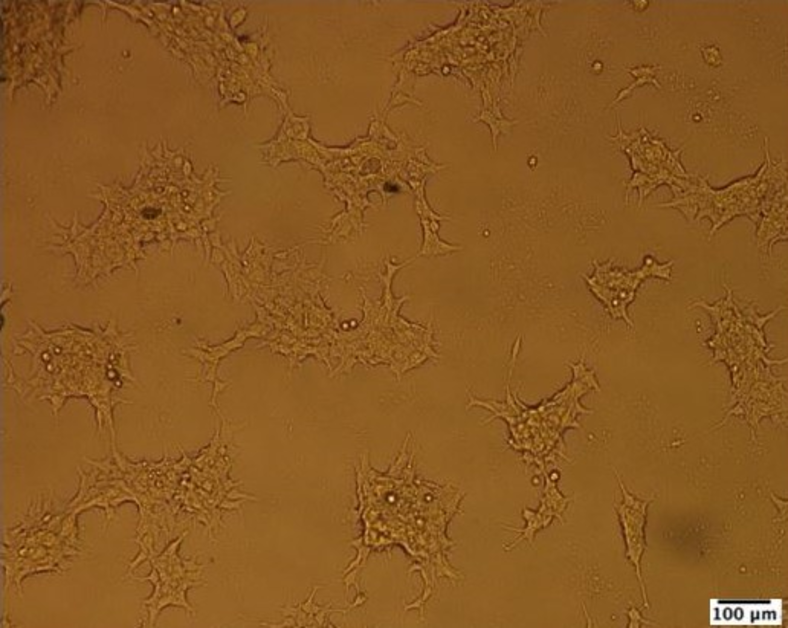

Supplement: S5 Fig — A. Anticancer activity (30%) of purified asparaginase at concentration of 30 μg ml-1 on U87 human malignant glioma cells. B. Untreated U87 human malignant glioma cells. C. Anticancer activity (55%) of purified L-asparaginase at concentration of 30 μg ml-1 on Huh7 hepatocyte carcinoma cells. D. Untreated Huh7 hepatocyte carcinoma cells. E. Cell viability (80%) of HEK293 human embryonic kidney cells with purified L-asparaginase at concentration of 30 μg ml-1. F. Untreated HEK293 human embryonic kidney. (ZIP) [file pone.0288620.s010.zip › S5D_Fig.tif]

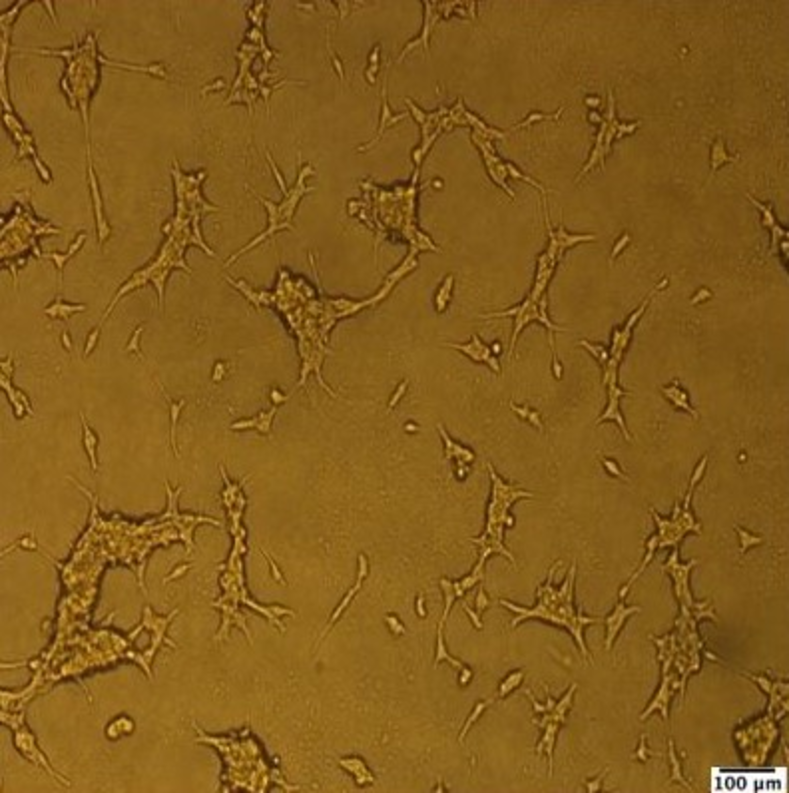

Supplement: S5 Fig — A. Anticancer activity (30%) of purified asparaginase at concentration of 30 μg ml-1 on U87 human malignant glioma cells. B. Untreated U87 human malignant glioma cells. C. Anticancer activity (55%) of purified L-asparaginase at concentration of 30 μg ml-1 on Huh7 hepatocyte carcinoma cells. D. Untreated Huh7 hepatocyte carcinoma cells. E. Cell viability (80%) of HEK293 human embryonic kidney cells with purified L-asparaginase at concentration of 30 μg ml-1. F. Untreated HEK293 human embryonic kidney. (ZIP) [file pone.0288620.s010.zip › S5E_Fig.tif]

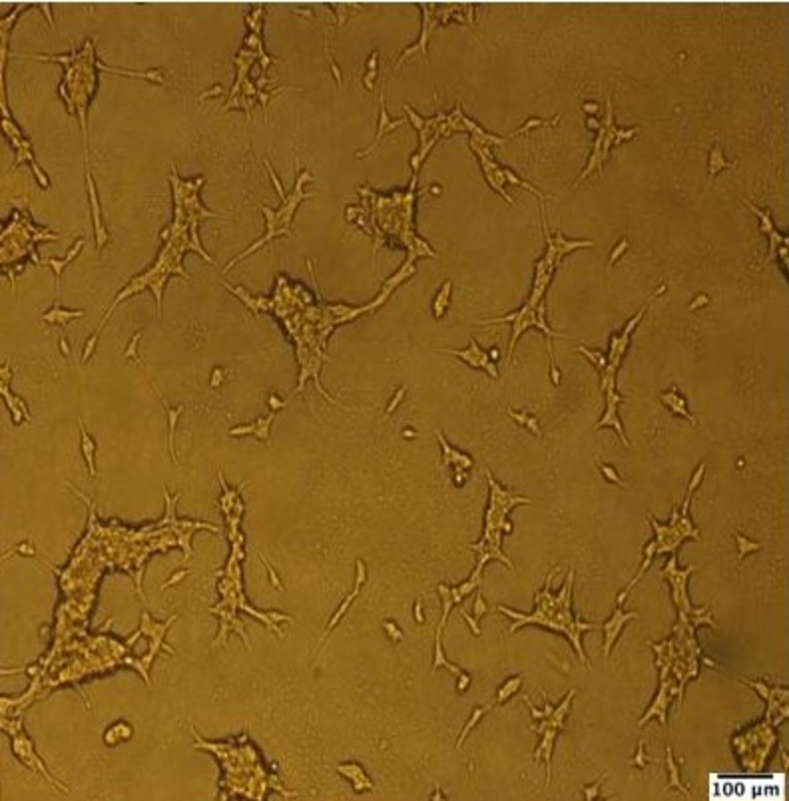

Supplement: S5 Fig — A. Anticancer activity (30%) of purified asparaginase at concentration of 30 μg ml-1 on U87 human malignant glioma cells. B. Untreated U87 human malignant glioma cells. C. Anticancer activity (55%) of purified L-asparaginase at concentration of 30 μg ml-1 on Huh7 hepatocyte carcinoma cells. D. Untreated Huh7 hepatocyte carcinoma cells. E. Cell viability (80%) of HEK293 human embryonic kidney cells with purified L-asparaginase at concentration of 30 μg ml-1. F. Untreated HEK293 human embryonic kidney. (ZIP) [file pone.0288620.s010.zip › S5F_Fig.tif]
